# Navigating diagnostic uncertainty in vulvar lichen sclerosus: When clinical and histopathologic findings diverge

**DOI:** 10.1016/j.jdin.2026.04.013

**Published:** 2026-04-27

**Authors:** Priyanka Kadam, Miriam K. Pomeranz, Shari R. Lipner

**Affiliations:** a Stony Brook Renaissance School of Medicine, Stony Brook, New York; b The Ronald O. Perelman Department of Dermatology, New York University Grossman School of Medicine, New York, New York; c Department of Dermatology, Weill Cornell Medicine, New York, New York

**Keywords:** clinical reasoning, gynecologic dermatology, lichen sclerosus, vulvar

*To the Editor:* A common clinical scenario arises when a patient presents with vulvar symptoms suggestive of lichen sclerosus (LS), with the biopsy demonstrating only nonspecific findings such as “lichenoid dermatitis” or “spongiotic dermatitis.” This discordance in clinico-pathologic correlation warrants practical guidance.

Nondiagnostic biopsies are common in clinically suspected LS. In a retrospective study of 115 patients with clinically diagnosed vulvar LS, among 39 who underwent biopsy, 13 were nondiagnostic or nonspecific.^1^ Both the 2023 International Society for the Study of Vulvovaginal Disease (ISSVD) consensus^2^ and the 2024 European S3 guideline^3^ affirm that biopsy is not required when clinical features are characteristic.

In clinical practice, several distinct scenarios require different approaches. When clinical findings clearly suggest LS but biopsy shows nonsclerotic lichenoid changes,^2^ the diagnosis should be presumed and treatment initiated. Conversely, when clinical findings are equivocal but histopathology demonstrates pathognomonic dermal sclerosis, the diagnosis is confirmed. The most challenging scenario occurs when both clinical and histopathologic findings are nonspecific.

In cases of clinical and histopathologic uncertainty, physicians must first evaluate for alternative or coexisting conditions. Spongiotic histopathology may reflect vulvovaginal candidiasis, which should be excluded with fungal culture or potassium hydroxide (KOH) preparation,^4^ or contact dermatitis, which warrants review of potential irritants and allergens.^5^ Vulvovaginal atrophy should be considered in postmenopausal patients, with trial of topical estrogen if appropriate.^4^ Importantly, lichenoid histopathology may reflect lichen planus (LP), which has distinct management implications. LP should be suspected when erosions or vaginal involvement are present.^5^ As the workup is undertaken, initiation of empiric treatment with ultra-potent topical corticosteroids (clobetasol propionate 0.05% ointment applied once daily for 12 weeks^3^) is reasonable, as these agents are also first-line therapy for LP and delay should be avoided.

A critical unanswered question concerns treatment duration in patients with suspected but unconfirmed LS whose symptoms resolve. Diagnostic delays are common,^5^ during which irreversible scarring may develop. The 2024 European guideline recommends lifelong maintenance therapy for confirmed LS to prevent scarring progression and reduce malignancy risk.^3^ Whether this applies to unconfirmed cases remains unclear, creating tension between the risk of disease progression if treatment is discontinued and the burden of indefinite therapy for a potentially incorrect diagnosis. Subsequent clinical appearance further guides management: development of architectural changes (labial resorption, clitoral hood fusion, introital narrowing) can help confirm the diagnosis depending on other features.^5^

The question of whether initial biopsy is necessary deserves reconsideration. In cases with classic clinical features, treatment should proceed regardless of histopathologic findings. However, biopsy remains valuable for distinguishing LP from LS and excluding squamous cell carcinoma. The decision requires individualization based on patient circumstances and preferences.

For patients with both nonspecific clinical and histopathologic findings, we recommend evaluation for alternative diagnoses, empiric treatment with close follow-up, documentation of therapeutic response, and shared decision-making regarding long-term management. Repeat biopsy rarely provides additional clarity. Referral to a vulvar specialist, where available, may be valuable for persistent diagnostic uncertainty. Future collaborative studies may help establish consensus recommendations for managing diagnostic uncertainty in suspected LS.

### Declaration of generative AI and AI-assisted technologies in the writing process

Generative AI was not used in the creation of this manuscript.

## Conflicts of interest

Dr Lipner has served as a consultant for Moberg Pharmaceuticals and BelleTorus Corporation. Dr Pomeranz is an author for UpToDate and a member of the scientific advisory committee of Proctor and Gamble. Ms Kadam has no conflicts of interest to declare.

## References

[1] McCarthy S., MacEoin N., O'Driscoll M., O'Connor R., Heffron C.C.B.B., Murphy M. (2019). Should we always biopsy in clinically evident lichen sclerosus?. J Low Genit Tract Dis.

[2] Day T., Selim M.A., Allbritton J.I., Scurry J., ISSVD Difficult Pathologic Diagnoses Committee (2023). Nonsclerotic lichen sclerosus: definition of a concept and pathologic description. J Low Genit Tract Dis.

[3] Kirtschig G., Kinberger M., Kreuter A. (2024). European guideline on the management of lichen sclerosus: part 2—treatment and follow-up. J Eur Acad Dermatol Venereol.

[4] Sobel J.D. (1997). Vaginitis. N Engl J Med.

[5] Schlosser B.J., Mirowski G.W. (2015). Lichen sclerosus and lichen planus in women and girls. Clin Obstet Gynecol.

